# Transverse plane pelvic rotation increase (TPPRI) following rotationally corrective instrumentation of adolescent idiopathic scoliosis double curves

**DOI:** 10.1186/1748-7161-5-18

**Published:** 2010-08-26

**Authors:** Marc A Asher, Sue-Min Lai, Brandon B Carlson, Jeffrey L Gum, Douglas C Burton

**Affiliations:** 1 Department of Orthopedic Surgery, Kansas University Medical Center, 3901 Rainbow Boulevard: Mail Stop 3017, Kansas City, KS 66160, USA; 2 Department of Preventive Medicine and Public Health, Kansas University Medical Center, 3901 Rainbow Boulevard, Kansas City, KS 66160, USA

## Abstract

**Background:**

We have occasionally observed clinically noticeable postoperative transverse plane pelvic rotation increase (TPPRI) in the direction of direct thoracolumbar/lumbar rotational corrective load applied during posterior instrumentation and arthrodesis for double (Lenke 3 and 6) adolescent idiopathic scoliosis (AIS) curves. Our purposes were to document this occurrence; identify its frequency, associated variables, and natural history; and determine its effect upon patient outcome.

**Methods:**

Transverse plane pelvic rotation (TPPR) can be quantified using the left/right hemipelvis width ratio as measured on standing posterior-anterior scoliosis radiographs. Descriptive statistics were done to determine means and standard deviations. Non-parametric statistical tests were used due to the small sample size and non-normally distributed data. Significance was set at *P *< 0.05.

**Results:**

Seventeen of 21 (81%) consecutive patients with double curves (7 with Lenke 3 curves and 10 with Lenke 6) instrumented with lumbar pedicle screw anchors to achieve direct rotation had a complete sequence of measurable radiographs. While 10 of these 17 had no postoperative TPPRI, 7 did all in the direction of the rotationally corrective thoracolumbar instrumentation load. Two preoperative variables were associated with postoperative TPPRI: more tilt of the vertebra below the lower instrumented vertebra (-23° ± 3.1° vs. -29° ± 4.6°, *P *= 0.014) and concurrent anterior thoracolumbar discectomy and arthrodesis (5 of 10 vs. 7 of 7, *P *= 0.044). Patients with a larger thoracolumbar/lumbar angle of trunk inclination or larger lower instrumented vertebra plus one to sacrum fractional/hemicurve were more likely to have received additional anterior thoracolumbar discectomy and arthrodesis (c = 0.90 and c = 0.833, respectively).

Postoperative TPPRI resolved in 5 of the 7 by intermediate follow-up at 12 months. Patient outcome was not adversely affected by postoperative TPPRI, whether or not it persisted.

**Conclusions:**

Our findings suggest that TPPRI is a decompensation caused by extension of the corrective thoracolumbar rotational load into the lumbosacral hemicurve below. As posterior instrumentation of adolescent idiopathic scoliosis becomes increasingly more effective in the transverse plane, postoperative TPPRI may become more widely noticed. This study provides some assurance that recompensation usually occurs, but that in either event TPPRI does not seem to affect clinical outcome.

## Background

Soon after the introduction of Cotrel-Dubousset instrumentation it became apparent that alignment changes in the transverse plane could result in imbalance and decompensation [1]. The continuing development of pedicle screw anchors, instruments to apply larger direct loads to the vertebra, and rigid anchor-rod connections has allowed ever-increasing control of spine position, including the transverse plane [2]. This was first apparent to us in the thoracolumbar/lumbar spine [3], and efforts were made to develop techniques that would maximize transverse plane deformity correction [4,5].

Eventually, we observed that postoperatively the whole pelvis sometimes appeared rotated in the same direction as the corrective rotational load to the thoracolumbar/lumbar spine [6]. This manifested itself clinically by different planes of shoulder and pelvis transverse plane rotation, with the shoulders generally in the mid-coronal plane and the pelvis rotated from the mid-coronal plane. On the standing posterior-anterior spine deformity radiograph the patient's hemipelvis shadow widths appeared asymmetrical. Although concerning to the surgeon and sometimes to the parents, this asymmetry usually seemed to resolve and did not appear to affect patient outcome. To our knowledge this transverse plane decompensation and recompensation have not previously been reported.

The purpose of this study was to answer 4 questions. First was how often clinically noticeable postoperative transverse plane pelvic rotation increase (TPPRI) occurred following primary posterior instrumentation and arthrodesis of double (Lenke 3 and 6) adolescent idiopathic scoliosis (AIS) curves utilizing direct thoracolumbar/lumbar rotational corrective loading. Second was if there were variables influencing the development of TPPRI. Third was how often it resolved. Fourth was if it affected clinical outcome.

## Methods

This study was approved by the Kansas University Medical Center Human Subjects Committee. It is a retrospective study based on a prospectively assembled cohort of consecutive patients, aged 10 through 20 years, receiving primary posterior instrumentation and arthrodesis for adolescent idiopathic scoliosis by one surgeon at one hospital. Additional inclusion criteria were Lenke 3 or 6 curves and direct application of corrective rotational loads through 3 or more lumbar pedicle screw anchors.

Posterior hybrid instrumentation (Isola, DePuy Spine, Raynham, Massachusetts) and arthrodesis were utilized. The surgical techniques have been described in detail [4,7]. Briefly, 3 or more thoracolumbar pedicle screw anchors, 2 of them in the lower instrumented vertebra, were utilized to provide direct rotational corrective loads to the thoracolumbar/lumbar spine. These loads were countered by indirect rotational corrective loads in the thoracic spine utilizing hook anchors and sometimes additional wire (or cable) anchors. One goal was to provide as nearly normal 3-dimensional alignment at the lower junctional region as possible. In addition, we strove to never instrument lower than L3 or its equivalent. Sequential 360° anterior thoracolumbar discectomy and arthrodesis, without anterior instrumentation, were sometimes utilized in an attempt to accomplish this. Although strict selection criteria for this additional surgery were not followed, generally larger, stiffer, and more rotated thoracolumbar/lumbar curves were selected.

**Figure 1:**
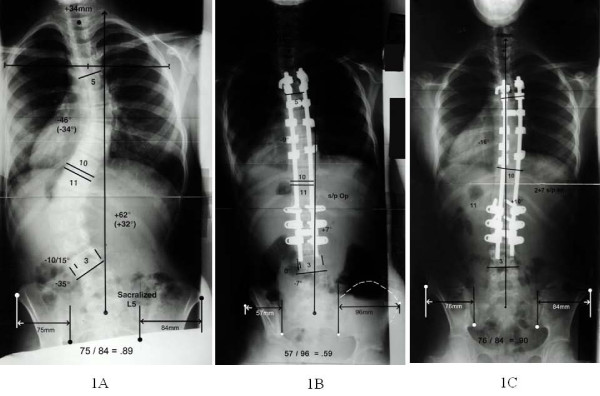
**Standing posterior-anterior radiographs of a patient with a type 6 curve**. The patient's postoperative transverse plane pelvic rotation (TPPR) increase resolved: preoperative (Fig. 1-A), postoperative (Fig. 1-B), and at 2 years, 7 months postoperative (Fig. 1-C). The method of measuring the left/right hemipelvis ratio, which directly relates transverse plane pelvis rotation, is illustrated. The landmarks are the inferior ilium at the sacroiliac joint medially and the anterior superior iliac spine laterally. The horizontal distance between these 2 points on each hemipelvis is expressed as a left/right hemipelvis ratio.

Stimulated by questions posed not only by the observation of TPPRI leading to this study but also by those related to the involvement of the pelvis in idiopathic scoliosis deformity evolution, we developed a method to quantify pelvic rotation. It utilizes clinically available standing posterior-anterior spine deformity radiographs [8]. The method depends upon the observation that transverse plane pelvis rotation of up to 20° is accurately reflected by a ratio of the iliac widths. The best-located and most reliable landmarks for this purpose were found to be the inferior ilium at the sacroiliac joint medially (SI) and the anterior superior iliac spine laterally (ASIS) (Figure 1). The horizontal distances between these 2 points on each hemipelvis are expressed as a left/right (L/R) hemipelvis ratio. The hemipelvis shadow becomes wider on the side to which the anterior aspect of the pelvis is rotated. Thus, the L/R hemipelvis ratio becomes less than 1 when the anterior pelvis is rotated to the right and greater than 1 when rotated to the left. Following the International Standards Organization Cartesian coordinate convention, rotation of the anterior pelvis to the right is clockwise (C) and to the left counterclockwise (CC) [9]. The L/R hemipelvis ratio is a reproducible measurement, with intra-observer agreement of 0.97 for 197 comparisons and inter-observer agreement of 0.88 for 48 AIS comparisons [10]. Determining the positioning reproducibility of the L/R hemipelvis ratio has not previously been done and is included in the current study.

Accurate conversion of the L/R hemipelvis ratio to degrees requires measurements not available on the clinically available radiographs. Because the L/R hemipelvis ratio is an indirect measure of pelvic rotation, we have defined postoperative transverse plane pelvic rotation increase (TPPRI) as beyond threshold L/R hemipelvis ratios associated with 5° or more rotation of the model of an adult female pelvis of known dimension [8]. These L/R hemipelvis ratios are ≤ 0.75 for clockwise (C) rotation and ≥ 1.35 for counterclockwise (CC) rotation, with a ratio of 1 being no rotation. A preoperative to postoperative change of 5° or more is an L/R hemipelvis ratio decrease of 0.25 or more for clockwise (C) rotation or increase of 0.35 or more for counterclockwise (CC) rotation. This is the definition of preoperative to postoperative increase. The definition for persistent TPPR increase is the same plus a TPPR L/R hemipelvis ratio of ≤ 0.75 or ≥ 1.35 at follow-up.

Radiographic pelvis rotation (L/R hemipelvis ratio) measurements were made on clinically obtained coronal plane posterior to anterior exposure spine radiographs taken on 91-cm (36-in) film at an 183-cm (72-in) tube to file distance. Patients were positioned so that a line connecting their heels would be parallel to the x-ray cassette. To standardize measurement direction, coronal plane radiographs of patients with left thoracic-right thoracolumbar (lumbar) were inverted (reversed) 180° horizontally.

R/L hemipelvis ratio measurements were made preoperatively, postoperatively, at intermediate follow-up (the one closest to 1 year postoperative), and at follow-up (the one closest to 2 years postoperative). In addition, R/L hemipelvis ratio measurements were made on any available measurable radiograph taken in the year before the preoperative radiograph, and the preoperative interval was recorded in months. These measurements were used to determine the positioning reliability of the R/L hemipelvis ratio measurement.

Clinical and radiographic data for variables possibly related to or influencing the development of TPPRI were gathered from clinic and hospital charts and radiographs. Previously recorded measurements were all confirmed or corrected, and added measurements were made. These variables included age and Risser sign at surgery, sex, curve pattern, curve orientation, operative case sequence, supplemental anterior discectomy and arthrodesis, upper and lower instrumented vertebra, and lower end vertebra. The lateral radiographs were taken into account when determining the Risser sign [11], and the curves were categorized using the Lenke classification [12]. Spine deformity radiographic measurements were recorded preoperative, postoperative, and at follow-up. The coronal plane radiographic measurements included balance (T1 offset from the center sacral line) and Cobb measurements of the thoracic, thoracolumbar/lumbar, lower instrumented vertebra to sacrum, and lower instrumented vertebra plus one below to sacrum curves. Supine, best effort anterior-posterior bend radiographs were used to determine curve mobility. The coronal plane tilt of the lower instrumented vertebra, lower instrumented vertebra plus one below, and the sacrum was measured. Transverse plane rotation of the thoracolumbar/lumbar apex vertebra preoperative and of the lower instrumented vertebra plus one below preoperative, postoperative, and at follow-up was measured in increments or 2.5° using the Perdriolle technique [13]. Transverse plane trunk rotation was measured as the angle of trunk inclination (ATI) [14] at the thoracic and thoracolumbar/lumbar levels at the preoperative, intermediate, and at follow-up intervals. Sagittal plane radiographic measurements were thoracic kyphosis (T2-T12), lumbar lordosis (T12-S1), and sacral slope.

Clinical outcome was based on the total score on an SRS HRQL questionnaire at follow-up. As several versions had been used as the series progressed, the results were normalized to a percent optimal score [15].

### Statistical Analysis

Descriptive statistics were done to determine means and standard deviations. The intra-class correlation coefficient (ICC) was used to calculate the positioning reliability of the R/L hemipelvis ratio utilizing preoperative radiographs taken within a year of each other. An ICC of ≥ 0.75 is considered to be excellent reliability [16].

Non-parametric statistical tests were used due to the small sample size and non-normally distributed data. Comparisons between independent groups were done using the Wilcoxon rank-sum test, and comparisons between pre- and postoperative and pre- and follow-up were done using the Wilcoxon signed-rank test. Both Wilcoxon tests used make statistical inferences about differences between medians. Comparisons of categorical variables between groups were done with the Fisher Exact or the Chi-square tests, as appropriate [17]. Significance was set at *P *< 0.05.

Logistic regression was used to explain differences in a combination of coronal and transverse plane variables for those with and without TPPRI as well as for the groups of patients who had posterior surgery only and those who had the sequential anterior discectomy and arthrodesis before the posterior instrumentation and arthrodesis [18]. The c statistic, which is equivalent to the Receiver Operating Characteristic (ROC), ranges from 0.5 to 1, where 0.5 corresponds to the model randomly predicting the response (namely receiving additional anterior thoracolumbar discectomy and arthrodesis), and a 1 corresponds to the model perfectly discriminating the response. The likelihood ratio test was used to obtain the significance level of variables of interest.

## Results

From 1993 through 2002, 23 patients, 10 with Lenke 3 curves and 13 with Lenke 6, were operated. Two of the patients with Lenke 3 curves did not have direct rotational loading of the thoracolumbar/lumbar screws, leaving 21 patients who met the study inclusion criteria. Of these 21 eligible patients, 17 (81%) had the necessary clinical and radiographic material to allow analysis. For these 17 patients the intermediate follow-up averaged 12 ± 3.0 months and the follow-up 30 ± 9.5 months.

The positioning reproducibility of the L/R hemipelvis ratio was calculated using those patients who had an earlier radiograph taken within a year before surgery. Eleven of the patients had sets of radiographs taken at a mean of 4.5 ± 1.57 months (range, 3 to 8 months) apart. The ICC was 0.75.

Seven of the 17 patients (41%) had postoperative transverse plane pelvis rotation increase (TPPRI) of 5° or more (Table 1). In all 7, rotation was in the direction of the directly applied thoracolumbar/lumbar corrective load. Their pre- and postoperative L/R SI-ASIS hemipelvis width ratios were 1.02 ± 0.116 and 0.70 ± 0.154, *P *= 0.0166. Compared to the no-TPPRI group, the change in preoperative to postoperative L/R ratios was 0.01 ± 0.138 vs. 0.32 ± 0.074, *P *= 0.0039. There were no differences between the 2 groups at intermediate follow-up or follow-up.

**Table 1:** Comparisons of left/right hemipelvis ratios for patients without and with Transverse Plane Pelvis Rotation Increase.

Study Group (N)	Preoperative	Postoperative		Intermediate (Int.) Follow-Up		Follow-Up (FU)	
	
	L/R Ratio	L/R Ratio	Pre L/R Minus Post L/R	L/R Ratio	Pre L/R minus Int. L/R	L/R Ratio	Pre L/R minus FU L/R
No TPPRI (10)	0.88 ± 0.128	0.88 ± 0.141	0.01 ± 0.138 **†**	0.92 ± 0.106	-0.03 ± 0.087	0.91 ± 0.073	-0.03 ± 0.132

TPPRI (7)	1.02 ± 0.116 **+**	0.70 ± 0.154 **+, ++**	0.32 ± 0.074 **†**	0.91 ± 0.176 **++**	0.12 ± 0.141	0.89 ± 0.152	0.13 ± 0.191

**† ***P *= 0.0039 + *P *= 0.0166 ++ *P *= 0.0156

**Figure 2:**
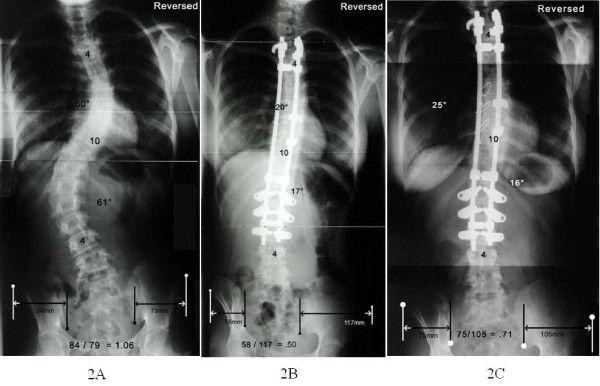
**Standing posterior-anterior radiographs of a patient with a type 3 curve**. The patient's postoperative TPPR increase did not resolve: preoperative (Fig. 2-A), postoperative (Fig. 2-B), and at 2 years, 3 months postoperative (Fig. 2-C). Patient has left thoracic-right thoracolumbar curve apexes, so the radiographs were viewed inverted horizontally (reversed).

The TPPRI resolved in 5 of the 7 patients by intermediate follow-up (Figure 1). One Lenke 3 and one Lenke 6 curve did not resolve. Their L/R hemipelvis ratios averaged 0.79 and 0.72 at intermediate follow-up and follow-up. Their preoperative L/R hemipelvis minus intermediate follow-up and follow-up L/R hemipelvis ratios averaged 0.23 and 0.30, respectively, representing the amount of persistent TPPRI (Figure 2).

Several demographic, phenotypic, and treatment variables possibly influencing postoperative TPPRI are listed in Table 2. Only the addition of supplemental, sequential thoracolumbar/lumbar anterior discectomy and arthrodesis was significant (*P *= 0.044). Although not significant, all 3 mirror image curves (left thoracic-right thoracolumbar/lumbar) had TPPRI (*P *= 0.051) and supplemental anterior surgery. Both patients with persistent TPPRI had mirror image curves (Figure 2).

**Table 2:** Independent demographic, phenotypic, and treatment variables possibly influencing postoperative TPPR Increase.

Variable	All Patients N = 17	No TPPR Increase N = 10	TPPR Increase N = 7	*P*
Age at surgery (years)	14.5 ± 2.39	15.1 ± 2.86	13.6 ± 1.22	ns^a^

Risser sign				

0	5	2	3	ns^b^
	
1	0	0	0	
	
2	6	4	2	
	
3	1	1	0	
	
4	3	1	2	
	
5	2	2	0	

Sex				

Females	15	8	7	ns^b^
	
Males	2	2	0	

Curve Pattern				

Lenke 3	7	4	3	ns^b^
	
Lenke 6	10	6	4	

Curve Orientation				

Rt. T - Lt TL/L	14	10	4	p = 0.051^b^
	
Lt. T - Rt. TL/L	3	0	3	

Case Sequence (1 through 17)	9 ±5.05	8.5 ± 4.93	9.7 ± 5.53	ns^b^

Supplemental, Sequential Anterior Discectomy and Arthrodesis, without Instrumentation				

No	5	5	0	p = 0.044^b^
	
Yes	12	5	7	

Upper Instrumented Vertebra (UIV): Lower Instrumented (LIV) & End Vertebra (LEV)				

UIV T3	14	10	4	ns^b^
	
T4 or T5	3	0	3	

LIV L2	2*	1*	1*	ns^b^
	
L3	15**	9**	6	

LEV L3	10***	6*	4*	ns^b^
	
L4	7**	4**	3	

* Two had 4 lumbar vertebrae ** One had 6 lumbar vertebrae *** Two had 4 lumbar vertebrae (one each in the no-TPPRI and TPPRI groups)

^a ^Two-tailed Wilcoxon rank-sum test

^b ^Fisher Exact or Chi-square test

Several coronal plane deformity variables possibly influencing TPPRI are shown in Table 3. The only significant preoperative difference was the tilt of lower instrumented vertebra plus one below. Compared to the no-TPPRI group, the TPPRI group was tilted more toward the left/apex of the thoracolumbar/lumbar curve, -23 ± 3.1° vs. -29 ± 4.6, *P *= 0.014. Postoperatively the only difference was in balance with the no-TPPRI group having better balance than the TPPRI group, 11 ± 18.5 mm vs. 29 ± 10.3 mm, *P *= 0.018. At follow-up the TPPRI group had rebalanced from 29 ± 10.3 mm to 7 ± 8.2 mm, *P *= 0.028. In addition to the measurements listed, the instrumented thoracolumbar/lumbar curve and the sacral tilt were measured, and no significant differences between the 2 groups at any interval were found. Preoperative sacral tilt averaged -4 ± 1.3° and -6 ± 3.3° in the no-TPPRI and TPPRI groups, respectively, and was not significantly different at any of the study intervals.

**Table 3:** Independent coronal plane radiographic deformity variables possibly related to postoperative TPPR Increase.

Variable	All Patients N = 17	No TPPR Increase (A) N = 10	TPPR Increase (B) N = 7	*P^a^*
Coronal Balance, mm; + left, - right				

Preoperative	17 ± 17.0	14 ± 15.8	22 ± 18.8	ns

Postoperative	18 ± 17.9	11 ± 18.5	29 ± 10.3	0.018

Follow-up (FU)	6 ± 10.8	5 ± 12.7	7 ± 8.2	ns

*P*^b ^Pre to Post: A, ns; B, ns Post to FU: A, ns; B, 0.028				

Thoracic Cobb °				

Preoperative	57 ± 9.2	58 ± 9.1	56 ± 9.9	ns

Bend	39 ± 6.3	37 ± 7.0	41 ± 4.9	ns

Postoperative	23 ± 7.9	23 ± 8.8	22 ± 6.9	ns

Follow-up	23 ± 6.9	22 ± 7.8	25 ± 5.5	ns

*P*^b ^Pre to Post: A, 0.005; B, 0.018 Post to FU: A, ns; B, ns				

Thoracolumbar/Lumbar Cobb °				

Preoperative	62 ± 6.3	60 ± 6.8	64 ±5.2	ns

Bend	32 ± 6.2	33 ± 5.9	31 ± 6.9	ns

Postoperative	17 ± 9.0	17 ± 10.8	17 ± 6.4	ns

Follow-up	16 ± 7.6	16 ± 9.2	17 ± 5.4	ns

*P*^b ^Pre to Post: A, 0.005; B, 0.018 Post to FU: A, ns; B, ns				

Lower Instrumented Vertebra (LIV) Tilt °; + right, - left				

Preoperative	-26 ± 7.8	-23 ± 7.1	-29 ± 7.9	ns

Postoperative	-3 ± 4.5	-2 ± 3.1	-4 ± 6.0	ns

Follow-up	-1 ± 2.9	-1 ± 3.4	-1 ± 2.2	ns

*P*^b ^Pre to Post: A, 0.005; B, 0.018 Post to FU: A, ns; B, ns				

LIV Plus One (vertebra below) Tilt °; + right, - left				

Preoperative	-26 ± 4.8	-23 ± 3.1	-29 ± 4.6	0.014

Postoperative	-7 ± 3.8	-7 ± 3.7	-7 ± 4.2	ns

Follow-up	-7 ± 5.4	-7 ± 6.5	-7 ± 3.7	ns

*P*^b ^Pre to Post: A, 0.005; B, 0.017 Post to FU: A, ns; B, ns				

Lower Instrumented Vertebra (LIV) to Sacrum Cobb: °				

Preoperative	21 ± 7.7	20 ± 6.8	22 ± 9.1	ns

Bend	5 ± 5.9 *	4 ± 4.6 **	5 ± 7.6	ns

Postoperative	0 ± 4.9	0 ± 3.3	0 ± 7.0	ns

Follow-up	-3 ± 3.8	-2 ± 3.6	-4 ± 3.8	ns

*P*^b ^Pre to Post: A, 0.005; B, 0.018 Post to FU: A, ns; B, ns				

LIV plus 1 (vertebra below) to Sacrum Cobb: °				

Preoperative	22 ± 4.7	20 ± 2.9	24 ± 5.9	ns

Bend	8 ± 4.5 *	6 ± 4.1 **	10 ± 4.5	ns

Postoperative	4 ± 4.0	5 ± 2.6	3 ± 5.4	ns

Follow-up	3 ± 5.2	5 ± 5.9	1 ± 3.4	ns

*P*^b ^Pre to Post: A, 0.005; B, 0.0018 Post to FU: A, ns; B, ns				

* n = 16 ** n = 9

Statistical comparisons only between the same patients

*P^a ^*= Wilcoxon rank-sum test *P*^b ^= Wilcoxon signed-rank test

The transverse plane trunk asymmetry variables possibly influencing TPPRI are shown in Table 4. Preoperatively there were no significant differences between the 2 groups. From pre- to postoperative, the lower instrumented vertebra plus one below rotation decreased and moved in the same direction as the thoracolumbar/lumbar rotation load, significantly in the TPPRI group: from 6 ± 4.3° to 2 ± 2.8°, *P *= 0.0313. At follow-up it had increased slightly to 3 ± 2.5°, but not significantly different from postoperative. From pre- to postoperative and post- to follow-up the lower instrumented vertebra plus one below rotation was unchanged in the no-TPPRI group.

**Table 4:** Clinical and radiographic transverse plane asymmetry variables possibly affecting postoperative TPPR Increase.

Variable	All Patients N = 17	No TPPR Increase (A) N = 10	TPPR Increase (B) N = 7	*P^a^*
Thoracic Angle of Trunk Inclination (Bunnell): °				

Preoperative	13 ± 4.14*	12 ± 2.3	14 ± 6.2****	ns

Intermediate Follow-up	7 ± 4.9 *	8 ± 3.6	4 ± 6.0****	ns

Follow-up	7 ± 4.0 **	8 ± 2.1***	5 ± 5.6****	ns

*P*^b ^= Pre to Post: A, 0.0313; B, 0.0625 Post to FU: A, ns; B, ns				

Thoracolumbar/Lumbar Angle of Trunk Inclination(ATI) (Bunnell): °				

Preoperative	15 ± 5.2	13 ± 4.4	17 ± 5.4	ns

Intermediate Follow-up	1 ± 1.9*	0 ± 1.8	2 ± 1.8	ns

Follow-up	2 ± 2.4**	1 ± 1.8***	3 ± 3.0****	ns

*P*^b ^= Pre to Post: A, 0.002; B, 0.0313 Post to FU: A, ns; B, ns				

Thoracolumbar/Lumbar Apex Vertebra Rotation (Perdriolle): °				

Preoperative	27 ± 6.8	24 ± 6.1	30 ± 6.6	ns

Lower Instrumented Vertebra +1 Rotation (Perdriolle): °				

Preoperative	6 ± 4.1	6 ± 4.1	6 ± 4.3	ns

Postoperative	5 ± 4.3	7 ± 4.1	2 ± 2.8	0.011

Follow-up	4 ± 3.3	5 ± 3.7	3 ± 2.5	ns
*P*^b ^= Pre to Post: A, ns; B, 0.0313 Post to FU: A, ns; B, ns				

* n = 16 ** n = 14 *** n = 8 **** n = 6

Statistical comparisons only between the same patients

*P^a ^*= Wilcoxon rank-sum test *P^b ^*= Wilcoxon signed-rank test

In the sagittal plane there were no significant differences between the no-TPPRI and TPPRI groups preoperative to follow-up. For all 17 patients the preoperative and follow-up values were T2 to T12 kyphosis 35 ± 12.8° and 32 ± 9.9°, T12 to sacrum lordosis -65 ± 12.3° and -64 ± 9.9°, and sacral slope 46 ± 9.3° and 45 ± 6.6°.

The c-statistics from a series of logistic regression models were used to search for coronal and transverse plane variables discriminating between the no-TPPRI and the TPPRI groups. No significant factors were observed in any of the models using the a-priori *P *value of < 0.05. The same was done to compare the posterior only and the sequential anterior then posterior groups. Two variables were shown to discriminate between the 2 groups: preoperative TL/L ATI (c-statistic: 0.90, *P *= 0.0062) and LIV+1 to sacrum Cobb (c-statistic: 0.833, *P *value = 0.0193). The respective preoperative TL/L ATI's were 11 ± 4.1° and 17 ± 4.8°. Their LIV +1 to sacrum curves were 18 ± 1.5° and 23 ± 4.9°.

At follow-up TPPRI resolved in 5 and persisted in 2. The radiographic and clinical variables of these 2 groups were carefully compared, and no apparent differences noted. The numbers were too small to make statistical comparisons meaningful.

At follow-up all of the patients had completed an HRQL questionnaire. The average scores for the no-TPPRI and TPPRI groups were 82% ± 12.2 and 90% ± 8.0, ns. The 2 patients with persistent TPPRI averaged 91% ± 5.7.

## Discussion

In this series of adolescent idiopathic scoliosis double curves we found postoperative transverse plane pelvic rotation increase (TPPRI) in 7 of 17, with resolution in 5 of the 7. Two of the variables studied were significantly associated with TPPRI. They were the addition of un-instrumented sequential anterior thoracolumbar/lumbar discectomy and arthrodesis and increased preoperative tilt of the vertebra below the lower instrumented vertebra. The occurrence of TPPRI and whether or not it persisted did not affect clinical outcome.

We interpret postoperative TPPRI to be a decompensation caused by extension of the corrective thoracolumbar/lumbar rotational load into the lumbosacral hemicurve below. This is supported by our finding that postoperatively the rotation of the vertebra below the lower instrumented vertebra in the direction of the thoracolumbar/lumbar curve (counterclockwise) had not changed in the no-TPPRI group, whereas in the TPPRI group it had decreased significantly, from 6° to 2°. Compared to the no-TPPRI group, the TPPRI group had significantly less postoperative rotation, 7° versus 2°. Transverse plane pelvis rotation increase re-compensated for 5 of 7 patients. Although we were unable to document the site of recompensation, at least partially because of the small number of patients, we believe that it occurred between the lower instrumented vertebra and the pelvis. The lack of postoperative to follow-up change in the coronal curves supports this.

Coronal plane decompensation also occurred in the TPPRI group and later resolved. Coronal plane recompensation appeared to occur at the disc below the lower instrumented vertebra. From postoperative to follow-up the tilt of the lower instrumented vertebra decreased from -4° to -1° in the TPPRI group while the tilt of the vertebra below was unchanged at 7°. This left the TPPRI group with an average of 6° disc wedge below the lower instrumented vertebra, which was the same for all 17 patients as a group.

Thus, both the transverse and coronal plane decompensation likely compensated in the junctional region just below the lower instrumented vertebra. We believe that recompensation was possible because this region had not been included in the instrumentation and arthrodesis.

Decompensation following transmitted rotation loading was noted soon after the introduction of Cotrel-Dubousset instrumentation [1,19]. Although the mechanism is the same as TPPRI, it was different as the rotation was transmitted from the thoracic spine, where it was corrective, to the upper lumbar spine, where it was deforming. Instrumenting this transitional zone both locked the deforming rotation in place and prevented recompensation through a mobile transition zone [1,19]. TPPRI, on the other hand, was occurring below the instrumented spine, leaving the transitional zone free to recompensate [20].

Although TPPRI has not been reported as far as we can tell, Dubousset has emphasized the concept of the pelvic vertebra, with six degrees freedom of motion between the hips and lumbosacral joint [21]. It is unlikely that TPPRI has gone unnoticed. However, it is difficult to document and quantify, does not appear to affect the patient's perceived quality of life, and usually resolves.

Double curve treatment results are seldom reported separately [15,22]. An exception is the series reported by Yeon et al. [23]. In 15 patients with Lenke 3 curves they tested the hypothesis that addition of anterior instrumentation to the anterior procedure would "more effectively correct and maintain normal coronal alignment in the distal unfused spine." The 7 patients treated with supplemental anterior instrumentation also had posterior instrumentation stopping at L3. In comparison, 6 of 8 treated without the addition of anterior instrumentation had posterior instrumentation to L4. Indeed, the tilt of L4 pre- and postoperative was the same for the 2 groups and was similar to ours without the addition of anterior instrumentation.

We believe TPPRI may have come to our attention at least partially because of our goals to never instrument below lumbar 3 or its equivalent and to leave the lower instrumented vertebra as normally aligned as possible. To accomplish this, we sought as complete correction of the thoracolumbar/lumbar curve as possible. Several measurements suggest that for the group this was pretty well accomplished: thoracolumbar/lumbar ATI improved from 16° to 1°, lower instrumented vertebra tilt from -26° to -3°, and the thoracolumbar/lumbar Cobb from 62° to 17°.

We realize the literature is not clear that instrumentation to L3, and the better instrumented curve correction necessary, is better than instrumentation to L4 in the long term. Our experience is the same as that reported by Islam et al. that the majority of previously operated scoliosis patients requiring surgical treatment of lower adjacent degeneration were originally instrumented and fused to L4 [24]. However, the L3-L4 motion segment is often at the junctional zone between curves, thus more mobile and less stable. Dubousset has recommended against stopping instrumentation and arthrodesis above the more mobile motion segment [21]. It will probably be a long time before the tradeoff between greater 3-planar correction and stopping at L3 or whether the less correction necessary with stopping at L4 is known.

We were disappointed that we could not develop more specific guidelines for adding supplemental anterior thoracolumbar/lumbar discectomy and arthrodesis in order to gain better correction. Patients with thoracolumbar ATI of ≤ 11° are less likely and those of ≥ 17° more likely to benefit from the supplemental surgery. The same can be said of those with a LIV +1 to sacrum Cobb angles of ≤ 18° and ≥ 23°. A possible relative variable that we could not quantify or evaluate is the "art" factor. The instrumentation sequence is complicated, and it is possible that it is not applied with equal effectiveness, even by the same surgeon in the same surgical environment.

It has been suggested that this TPPRI phenomenon is unique to the brand of instrumentation used. We have no material to make a direct comparison. However, we believe the finding is related to the effectiveness of direct spine rotation and that the type of instrumentation used to achieve it is immaterial.

Our study is open to several criticisms. Our method of transverse plane pelvis rotation measurement is not precise. Anticipatory CT imaging is unlikely for a new observation and unjustified given the apparent lack of clinical importance of TPPRI. Our method does allow useful quantification to be made from clinically available radiographs. As documented in this and our previous study, the intra-observer, inter-observer, and positioning reliabilities are good. Our method also cannot compensate for intrinsic pelvic asymmetry. For 2 reasons these shortcomings do not appear to be detrimental to this study. First, the baseline preoperative measurement was nearly neutral. Second, the L/R hemipelvis ratios were also compared for change. And, constrained patient positioning, especially if it included the pelvis, would possibly/probably mask this largely temporary transverse plane pelvic decompensation. Evolving, clinically practical three-dimensional imaging technology will make it possible to address these criticisms in future series.

These findings have to be considered preliminary because of the small number of patients operated. The observed marginal significance in the preoperative TL/L ATI's when comparing the posterior only with the anterior then posterior surgery groups in part may be due to the small number of patients in the study. To confirm the observed difference of the preoperative TL/L ATI's (11 ± 4.1° and 17 ± 4.8°) between the 2 groups as being significant at an alpha level of 0.05 (2-sided) and a statistical power of 80%, one must have at least 10 patients per group. To account for multiple comparisons (or an alpha of 0.01; 2-sided), one must have at least 15 patients per group at the same level of an 80% statistical power.

## Conclusions

We have reported, we believe for the first time, postoperative transverse plane pelvic rotation increase (TPPRI) in the direction of directly applied rotationally corrective thoracolumbar/lumbar loads during instrumentation and arthrodesis of double AIS curves. Clinically noticeable postoperative TPPRI of approximately 5° or more in the direction of the thoracolumbar/lumbar corrective rotational load was present in 7 of 17 patients with double curves. The only associated variables were concurrent anterior thoracolumbar discectomy and arthrodesis and tilt of the vertebra below the lowest instrumented vertebra. The postoperative TPPRI resolved in 5 of the 7 by the intermediate follow-up at an average of 12 months postoperative. Postoperative TPPRI, whether or not it resolved, did not adversely affect clinical outcome.

## Competing interests

MAA has received royalties and stock options for personal or professional use from a commercial party related directly or indirectly to the subject of this manuscript. DCB has a consulting agreement with a commercial party. The other authors do not have conflicting financial interests.

## Authors' contributions

MAA conceived the study, participated in its design and helped draft the manuscript. SML participated in the design of the study, statistical analysis and draft of the manuscript. BCC participated in the data gathering, statistical analysis and draft of the manuscript. JLG participated in the design of the study and collection of data. DCB participated in the design of the study, collection of data, and draft of the manuscript. All authors read and approved the final manuscript.
